# Diagnosing comorbidity in psychiatric hospital: challenging the validity of administrative registers

**DOI:** 10.1186/1471-244X-13-13

**Published:** 2013-01-08

**Authors:** Terje Øiesvold, Mary Nivison, Vidje Hansen, Ingunn Skre, Line Østensen, Knut W Sørgaard

**Affiliations:** 1 Institute of Clinical Medicine, Faculty of Health Sciences, University of Tromsø, and Division of general psychiatry, Nordland hospital, Bodø, Norway; 2 Clinic for substance abuse and specialized psychiatry, University Hospital of Northern Norway, Tromsø, Norway; 3 Institute of Clinical Medicine, Faculty of Health Sciences, University of Tromsø, and Division of general psychiatry, University Hospital of Northern Norway, Tromsø, Norway; 4 Department of Psychology, Faculty of Health Science, University of Tromsø, Tromsø, Norway; 5 Division of general psychiatry, Nordland hospital, Bodø, Norway; 6 Institute of Clinical Medicine, Faculty of Health Sciences, University of Tromsø, and Division of general psychiatry, Nordland hospital, Bodø, Norway

**Keywords:** Comorbidity, Psychiatric hospital, Psychiatric case register

## Abstract

**Background:**

This study will explore the validity of psychiatric diagnoses in administrative registers with special emphasis on comorbid anxiety and substance use disorders.

**Methods:**

All new patients admitted to psychiatric hospital in northern Norway during one year were asked to participate. Of 477 patients found eligible, 272 gave their informed consent. 250 patients (52%) with hospital diagnoses comprised the study sample. Expert diagnoses were given on the basis of a structured diagnostic interview (M.I.N.I.PLUS) together with retrospective checking of the records. The hospital diagnoses were blind to the expert. The agreement between the expert’s and the clinicians’ diagnoses was estimated using Cohen’s kappa statistics.

**Results:**

The expert gave a mean of 3.4 diagnoses per patient, the clinicians gave 1.4. The agreement ranged from poor to good (schizophrenia). For anxiety disorders (F40-41) the agreement is poor (kappa = 0.12). While the expert gave an anxiety disorder diagnosis to 122 patients, the clinicians only gave it to 17. The agreement is fair concerning substance use disorders (F10-19) (kappa = 0.27). Only two out of 76 patients with concurrent anxiety and substance use disorders were identified by the clinicians.

**Conclusions:**

The validity of administrative registers in psychiatry seems dubious for research purposes and even for administrative and clinical purposes. The diagnostic process in the clinic should be more structured and treatment guidelines should include comorbidity.

## Background

Psychiatric comorbidity is a prevalent phenomenon and remains a challenge for the effective delivery of mental health services. Recent community surveys show that among those with a psychiatric disorder the lifetime prevalence of more than one diagnosis is about 50% [1]. The highest rates of comorbidity are observed between anxiety and affective disorders [1], and affective, anxiety and substance use disorder often occur together [2,3]. The presence of comorbid disorders is associated with a significantly higher rate of help seeking [4-6].

To what extent psychiatric case registers or administrative registers reflect this comorbidity is not known. Since the 1960s, psychiatric case registers have been regarded as important epidemiological research tools for estimating treated incidence, prevalence and patterns of care [7]. With the development of new and better information and communication technologies, their importance is expected to increase [8,9]. Much of the utility of a psychiatric case register, however, will depend on the validity of the psychiatric diagnoses [10]. Byrne et al. [11] in their review conclude that relatively little high-quality work exists into systematically measuring the diagnostic data validity of registers for research purposes. Almost no studies (1 out of 14) performed anything else than case note reviews to assess validity. Only two of the studies reviewed stated that the register diagnoses were blinded to the researchers and inter-rater reliability testing was only performed in three of the studies.

Thus, there is a need for studies using more stringent methodological approaches to estimate the validity of case register diagnoses. In our study a structured diagnostic interview was performed on all new patients consecutively admitted to psychiatric hospital, comparing these diagnoses with those given by the clinicians. In a previous paper we have focused on the underdiagnosing of bipolar disorder [12]. In this paper we will present the results with regard to comorbid anxiety and substance use disorders.

## Methods

### Design and participants

The North-Norwegian study on first-time admitted patients to psychiatric hospital (FINN-study) is a prospective cohort study on treated incidence, utilization, and outcome in a one-year period and a 12-month follow-up period. The University Hospital in Northern Norway in Tromsø, and Nordland Hospital in Bodø, participated. All admissions to psychiatric hospitals in a region with a population of about 500 000 people are administered by these two hospitals. There are 14 community mental health centres in the region. The psychiatric services in Northern Norway are fully described elsewhere [13].

Included in the study were patients between 18 and 65 years of age who had no previous admissions to the participating hospitals and who gave written informed consent to participate. Exclusion criteria were: Lack of language competency and cognitive impairment such as dementia, serious mental retardation or other mental incapacities preventing the individual from giving an informed written consent. Further, The Regional Ethics Committee for Medical Research required that a patient be given at least 24 hrs after admission to consider participation, and hence patients who were discharged less than 3 days after admission had to be excluded. Thus comprehensive in/out interviews were unfeasible for short-stay patients. Of 674 first-time admitted patients, 477 patients were found eligible for participation. 272 patients gave their informed consent, and of these 250 patients (52%) with hospital diagnoses comprised the study sample.

### Data collection

Diagnoses were assessed by means of the Mini International Neuropsychiatric Interview PLUS (M.I.N.I.PLUS) [14] Norwegian version 5.0.0. [15]. M.I.N.I. was developed in Europe and USA as a short diagnostic instrument for generating DSM-IV criteria diagnoses convertible to ICD.10 diagnoses [16]. The M.I.N.I.PLUS is an extended version of the M.I.N.I. that includes information on specific phobias and has an expanded psychosis module. The M.I.N.I.PLUS is built up of 15 modules corresponding to diagnostic categories and collects information along 23 axis-I problem areas in relation to past and current symptoms. The interviews were carried out by psychiatric nurses, psychologists, graduate students in psychology, a resident doctor and a psychiatrist. Except for the two students, all had extensive clinical experience and none had therapeutic or other relations to the patients. The interviewers underwent systematic training and consecutive reliability checks using videotaped interviews. The interview was performed as soon as possible after admission when the patient was found eligible to participate in an interview and had given written consent.

An experienced psychologist (I. Skre), who has studied the validity and reliability of psychiatric diagnoses during two decades [17,18], will in the following will be referred to as the expert. She was not employed at the participating hospitals and she determined the diagnoses on the basis of the M.I.N.I. PLUS interviews and retrospective inspection of the patients’ records. The expert was blind to the hospital diagnoses. First, the M.I.N.I.PLUS schedule, including notes made by the interviewer, was reviewed and scored according to the ICD-10 criteria as they appear in ICD-10 Diagnostic Criteria for Research [19]. In cases where the information given in the interview was meagre, lacking or contradictory, additional information about the patient was sought from the hospital records: (1) the referral letter applying for admission, which accompanies all admissions to psychiatric hospitals in Norway, (2) the notes written by the receiving medical doctor at the hospital, and (3) when involving an involuntary admission, the notes written by the specialist in psychiatry/psychology who did the formal evaluation. In order to keep the expert blind to the hospital diagnoses and the referring physician’s tentative diagnosis, the information was extracted from the patient’s file and read aloud to the expert by an assistant. The assistant was instructed to omit all material concerning diagnostic evaluations. The following information was extracted from these documents: (1) the symptoms and behaviour of the patient in the days and hours immediately prior to hospitalization, (2) the symptoms and behaviours observed and described by the receiving medical doctor and/ or the specialist in psychiatry/psychology at the hospital. In some cases, when suspecting an organic mental disorder, any documentation of results from brain imaging and neuropsychological tests were used.

In accordance with the ICD-10, a diagnostic hierarchy was employed only when exclusion criteria were explicitly given in the diagnostic manual. When assigning more than one diagnosis, the diagnoses were listed in the following order: The first or primary diagnosis was always the disorder from which the behaviours or symptoms stemmed which had resulted in hospitalisation. Following the main diagnosis were additional disorders diagnosed in the patient, most often anxiety or somatoform disorders. Finally, diagnoses for harmful use of or dependence on psychoactive substances were assigned.

Hospital clinicians are obliged to use the ICD-10 criteria and to make a diagnostic evaluation in the discharge letter which is routinely sent to the patient’s GP. The hospital diagnosis is based on clinical interviews and observations made during the hospital stay. Interviews with relatives may be used as well as rating scales and structured interviews, but this is uncommon. The clinician’s diagnoses are given in the discharge letter from the hospital. Usually, what is considered the main disorder causing hospitalization is entered first, as the primary diagnosis, and additional diagnoses, if assigned, are entered subsequently. The discharge letter is the hospital’s summary of diagnostic evaluations, symptoms and behaviours which have been the focus for treatment, and which the hospital finds it important to communicate to the patient’s GP, or other primary or secondary case manager. Thus any clinical significant symptoms observed and addressed during hospital stay should be mentioned at discharge.

The Regional Ethics Committee of Northern Norway approved the study.

### Statistics

Descriptive statistics were used to present sample characteristics and the frequencies of the different diagnoses given by the clinicians as well as by the expert. Kruskall-Wallis and chi-square statistics were used to assess possible bias in the study sample. Cohen’s kappa (к) was used to estimate degree of agreement between expert and clinical diagnoses. According to the guidelines of Landis and Koch [20], a kappa agreement < .20 is poor, .21 - .40 is fair, .41 - .60 is moderate, .61-.80 is good and >.81 is almost perfect. SPSS 16.00 was used in the statistical analyses.

## Results

### The study sample

The study sample was comprised of 250 patients. As can be seen from Table 1 the mean age was 40.4 years, 111 (44.4%) were females, 71 (28.4%) were married or cohabiting, 241 (96.4) were of Norwegian ethnicity, 74 (29.6%) had paid work, 60 (24.0%) were voluntarily admitted and mean length of stay was 37.1 days. Participants were younger; more often had paid work, were more often voluntarily admitted and were admitted for longer lengths of stay than nonparticipants.

**Table 1:** Characteristics and possible biases (Kruskal-Wallis & Chi-square (X^2^)) of the sample (N=250)

		***Excluded***	***Included,no***	***Participated***	***X***^***2***^***/p***
		***N = 197***	***participation***		
		***(N,*%*)***	***N = 227***	***N =250***	
			***(N,*%*)***	***(N,*%*)***	
Age		41.4 (sd 21.0)	44.2 ( sd 19.9)	40.4 (sd 15.2)	Kruskal-Wallis, ns
Age groups	> 39 yrs	122 (61.9%)	113 (49.8%)	139 (55.6%)	X ^2^ = 26.64, p =.000
	40 - 59	33 (16.8%)	64 (28.2%)	81 (32.4%)	
	60 +	42 (21.3%)	50 (22.0%)	33 (12.0%)	
Females		92 (46.7%	97 (42.7%)	111 (44.4%)	ns
Married		51 (26.2%)	48 (21.3%)	71 (28.4%)	ns
Norwegian ethnicity		136 (69%)	215 (94.7%)	241 (96.4%)	X^2^ = 92.34, p = .000
Employment/income	Paid work	34 (17.3%)	50 (22.0%)	74 (29.6%)	
	National insurance benefits	121 (61.4%)	128 (56.4%)	116 (46.9%)	X^2^ = 12.81, p = .012
	Other	42 (21.3%)	49 21.6%)	60 (24.0%)	
Voluntary admission		124 (63.6%)	138 (61.1%)	193 (78.8%)	X^2^ = 19.89, p = .000
Length of first stay		11.6 (sd 19.6)	31.0 (sd 37.8)	37.1(sd 48.3)	Kruskal Wallis p < .001

### Degree of agreement between expert’s diagnoses and clinicians’ diagnoses

The expert gave a mean of 3.4 diagnoses while the clinicians only gave 1.4. The most common diagnoses given by the expert were drug and alcohol abuse (57%), major depression without psychosis (49%) and anxiety disorder (48%). Only major depression without psychosis was common among the diagnoses made by clinicians (51%). The agreement, as estimated by the kappa statistics, between the expert and the clinicians ranges from poor to good for the different diagnostic groups. Generally the agreement was better when comparing all diagnoses given opposed to comparing only the first diagnosis given [12].

Especially concerning anxiety disorders (F40-41) the agreement was poor (kappa = 0.12). While the expert gave an anxiety disorder diagnosis to 122 patients, the clinicians diagnosed anxiety disorder in only 15 of these, and altogether in 17 patients. Very few patients were assigned an anxiety disorder as their primary diagnosis, only 3 by the expert and 2 by the clinicians. The agreement was fair concerning substance use disorders (F10-19) with a kappa value of 0.27. 144 patients were diagnosed with substance use disorder by the expert, and for four patients this was their primary diagnosis. The corresponding figures regarding the clinicians were 45 and 22 patients respectively (Table 2).

**Table 2:** Frequency and degree of agreement (Cohens kappa/κ) between expert and clinician with regard to substance use disorder and anxiety disorder; all diagnoses set and primary diagnoses only (N=250)

	**All diagnoses**					**Primary diagnoses**				
		expert	clin.				expert	clin.		
	N^a^	n^b^	n^c^	n^d^	κ	N^a^	n^b^	n^c^	n^d^	κ
		(%)	(%)				(%)	(%)		
Substance use disorder (F10-19)	145	144	45	44	.27*	24	4	22	2	.20*
		(57)	(18)				(2)	(9)		
Anxiety disorder (F40-41)	124	122	17	15	.12*	4	3	2	1	.39*
		(48)	(7)				(1)	(1)		

N^a^ = number of patients given the diagnosis either by expert or by clinician.

n^b^ = number of patients given the diagnosis by expert.

n^c^ = number of patients given the diagnosis by a clinician.

n^d^ = number of patients given the diagnosis by both expert and clinician.

* P<0.001.

Tables 3 shows the same results in some more detail with regard to anxiety disorders (F40 – F41). Only 8 patients were given the same anxiety disorder diagnosis by the clinicians as by the expert.

**Table 3:** Anxiety disorder diagnoses (F40-41) set by the clinicians (N=122)

	**Expert**	**Clinicians**	
		**F40-41**	**The same diagnosis as the expert**
Agoraphobia F40.0	79	12	5
Social phobia F40.1	54	5	1
Specific phobias F40.2	24	4	0
Panic disorder F41.0	11	1	0
GAD F41.1	23	3	2

### Concurrent comorbidity of both anxiety disorder (F40-41) and substance use disorder (F10-19)

Seventy-six patients got both an anxiety disorder and a substance use disorder diagnosis from the expert. Seventy-five of these patients got additional diagnoses. As the primary diagnosis 93% had an affective disorder (F30-39) of which 26 (37%) were bipolar. One patient had schizophrenia and 4 patients schizoaffective disorder as the primary diagnosis. Only two of these patients were diagnosed with concurrent anxiety and substance use disorders by the clinicians (Table 4).

**Table 4:** Concurrent comorbidity of anxiety (F40-41) and substance use disorder (F10-19) diagnosed by the clinicians (N=76)

	**Expert**	**Clinicians**		
		**F10-19 and F40-41**	**F10-19**	**F40-41**
All patients	76	2	23	3
Affective disorder F30-39	70 (93%)	1	18	2
Bipolar disorder F31	26 (35%)	1	4	1
Sciz. spectrum F20(1) & F25(4)	5 (7%)	0	0	0

## Discussion

This study casts doubt upon the validity of administrative registers concerning comorbidity of anxiety and substance use disorder. To diagnose anxiety in particular, seems to be neglected; the clinicians gave such a diagnosis to only 17% of those identified with an anxiety disorder (F40-41) by the expert. Concerning substance use disorders (F10-19) the picture is somewhat better with 31% identified by the clinicians. Regarding concurrent comorbidity of anxiety and substance use disorder, this was to a very small degree identified by the clinicians; only in two of 76 patients. Almost all these patients had a primary affective disorder.

Not many studies have looked at the quality of administrative registers with regard to comorbidity. Only one of the studies reviewed by Byrne et al. [11] did so, finding that comorbidity was under-reported as in our study [21]. In an investigation of the Danish Psychiatric Register, Hansen et al. [22] reported underdiagnosing of substance use disorder by nearly 50%. Further, several studies reported that comorbid anxiety are being missed in clinical practice [23,24].

The question is raised whether comorbidity actually is an artefact of current diagnostic systems [25]. There is a marked symptom overlap between at first hand affective and anxiety disorders which could be a source of diagnostic unreliability and a dimensional classification system based on shared features of anxiety and mood disorders have been proposed [26]. In clinical practice a dimensional approach to treatment is common and a formal diagnosis is as a rule not given until discharge. It could be that the clinicians are aware of comorbid anxiety and substance use without diagnosing it formally [27]. One could imagine a kind of ethical considerations of not stigmatizing the patient with too many diagnoses giving only those who primarily brought the patient into hospital. However, Zimmerman and Chelminski [24] report underrecognition of anxiety disorders, for which the patients want treatment, in psychiatric outpatients with a principal diagnosis of major depression.

There could be several other reasons for this underdiagnosing of comorbidity. Treatment guidelines commonly only relate to specific diagnostic groups like affective disorders or anxiety disorders, and not to comorbid conditions [28]. This could make the clinicians less aware of comorbid conditions. The relation between co-occuring substance and anxiety disorders has not received much attention and is generally poorly understood. The diagnostic challenge in relation to individuals with current substance use disorders has been to devise diagnostic criteria and measurement techniques that differentiate between intoxication and withdrawal symptoms and the symptoms of psychiatric disorders. Many of the symptoms of intoxication and withdrawal from alcohol and other substances resemble the symptoms of mood and anxiety disorders [29]. Diagnostician using a structured interview routinely asks control questions that delineates between transient anxiety caused by substance withdrawal, and chronic anxiety symptoms. The results could also be due to the general phenomenon that clinicians rely on a limited number of heuristic principles which in some instances may lead to severe and systematic errors [30,31]. We believe clinicians are more apt to use a heuristic top-down approach when they diagnose patients, i. e. not asking about other symptoms when the patient presents with depression. The expert employing data from a structured clinical interview, however, employs a bottom-up approach in the diagnostic process, i. e. asking questions which at first seem irrelevant. The risk of misclassification is supposed to be higher using the top-down diagnostic approach and relying on the diagnostic manual to confirm a clinical impression rather than to openly screen for alternative or additional diagnoses. Lack of relevant information in the patients’ records is shown to be a general phenomenon affecting all diagnostic groups [32].

The importance of correctly diagnosing comorbidity in clinical practice should be emphasized. Several studies have shown that psychiatric comorbidity is associated with a significantly increased probability of treatment and that comorbidity can be regarded as an index of a more severe course and outcome of mental disorders [1]. Comorbidity is associated with more severe psychiatric symptoms, more functional disability, longer illness duration, less social competence, and higher service utilization [33]. Furthermore, patients with affective disorder and comorbid anxiety and substance abuse, show less adherence to pharmacological treatment [34] and need more specialized treatment [35]. It is shown that such comorbidity is associated with suicidality in mood disorders [36]. This underpins the importance of correctly diagnosing comorbid conditions in the clinic. The psychiatric evaluation and diagnoses given at discharge from psychiatric hospital will follow the patient and is guiding for the treatment the patient will receive from GP’s and psychiatric personnel in the community.

Concerning administrative consequences, not diagnosing comorbidity represents an undercommunication of the burden these patients represent to the health care system and consequently gives the wrong signals concerning how to develop necessary services. Misleading medical statistics may cause spurious comparisons during the planning and evaluation of treatment for patients [1]. Further, our findings suggest that register diagnoses are dubious for research purposes when it comes to comorbid psychiatric diagnoses. This is in accordance with the investigations of Baca-Garcia et al. [37,38] and McConville et al. [39].

Our study comprises only 250 patients, and only first time admissions, so the generalizability of the findings could be questioned. It could be argued that new patients are more difficult to diagnose making the diagnostic validity of registers including all patients, better. Compared with the studies reviewed by Byrne et al. [11] on the diagnostic validity of administrative registers in psychiatric research, our study has some advantages strengthening the validity of the results. First, a structured diagnostic interview was performed, adding information from the records when necessary, and the clinical diagnoses were blind to the expert. On the other hand, the expert never actually saw the patient. Thus the observations, scorings and case notes could have been evaluated otherwise if the expert had observed the patient directly. The greatest possible caveat here is that signs and symptoms may have been missed or misinterpreted. However the expert only scored a symptom as present if there was given a description of overt behaviour or citations from the patient in either the interview protocol or in the hospital records. Furthermore there is always a risk that diagnoses based on an interview which screens for all psychiatric symptoms may be overinclusive. This possible bias may result both from a “yes-saying” response style of the patient, and from a tendency of the interviewer to put weight on positive answers about signs and symptoms that are not clinically significant. Thus, there is a risk that the high number of diagnoses given by the expert is a result of response bias and scoring bias. However, we do not believe that this bias will disturb the main findings. Structured interviews are shown to be better than unstructured traditional diagnostic assessment [40,41], and combining structured interviewing with a review of the medical records appears to produce more accurate primary diagnoses and to identify more secondary diagnoses than routine clinical methods or a structured interview alone [42]. The studies reviewed by Byrne et al. [11], where only case notes were checked and no new information added, should be regarded more as reliability studies than validity studies. Second, in our study the clinicians’ diagnoses were blind to the expert thus avoiding bias in either direction. A weakness in this study may be that formal inter-rater reliability testing was not done among the interviewers, however, there were organized discussions among them, also on selected videotaped cases. Inter-rater reliability can be low even if diagnoses are determined by researchers as found by Cheniaux et al. [43]. However, to counter this, diagnoses were not formulated by the interviewers, but by one experienced researcher.

## Conclusions

Even if the nosological status of the comorbidity concept is by no means clear [25,44] the importance of correctly diagnosing comorbidity should be emphasized and the results from this study tells us that much work needs to be done in the clinic in structuring the diagnostic process, and in developing treatment guidelines for comorbid conditions [41,45,46]. The validity of psychiatric administrative registers concerning comorbidity, seems dubious for research purposes as well as for administrative and clinical purposes.

In a previous paper we have reported that this is even the case especially for bipolar disorder [12]. When it comes to psychotic disorders such as substance use induced psychosis and schizophrenia, the validity seems satisfactory.

## Competing interests

The authors declare that they have no competing interests.

## Authors’ contribution

TØ participated in designing the study and writing of the protocol, collected data, managed the literature searches and analyses, undertook statistical analyses and wrote the manuscript. MN participated in designing the study and writing the protocol, collected data, undertook statistical analyses and contributed to the final manuscript. VH participated in designing the study and writing of the protocol and contributed to the final manuscript. IS collected data and contributed to the final manuscript. LØ collected data, undertook statistical analyses and contributed to the final manuscript. KWS participated in designing the study and writing of the protocol, collected data, undertook statistical analyses and contributed to the final manuscript. All authors read and approved the final manuscript.

## Pre-publication history

The pre-publication history for this paper can be accessed here:

http://www.biomedcentral.com/1471-244X/13/13/prepub
